# A method to identify and validate mitochondrial modulators using mammalian cells and the worm *C. elegans*

**DOI:** 10.1038/srep05285

**Published:** 2014-06-13

**Authors:** Pénélope A. Andreux, Laurent Mouchiroud, Xu Wang, Virginija Jovaisaite, Adrienne Mottis, Sabrina Bichet, Norman Moullan, Riekelt H. Houtkooper, Johan Auwerx

**Affiliations:** 1 Laboratory of Integrative and Systems Physiology, École Polytechnique Fédérale de Lausanne, Switzerland; 2 Laboratory Genetic Metabolic Diseases, Academic Medical Center, University of Amsterdam, Amsterdam, The Netherlands; 3 Current address: Amazentis SA, Lausanne Switzerland.

## Abstract

Mitochondria are semi-autonomous organelles regulated by a complex network of proteins that are vital for many cellular functions. Because mitochondrial modulators can impact many aspects of cellular homeostasis, their identification and validation has proven challenging. It requires the measurement of multiple parameters in parallel to understand the exact nature of the changes induced by such compounds. We developed a platform of assays scoring for mitochondrial function in two complementary models systems, mammalian cells and *C. elegans.* We first optimized cell culture conditions and established the mitochondrial signature of 1,200 FDA-approved drugs in liver cells. Using cell-based and *C. elegans* assays, we further defined the metabolic effects of two pharmacological classes that emerged from our hit list, i.e. imidazoles and statins. We found that these two drug classes affect respiration through different and cholesterol-independent mechanisms in both models. Our screening strategy enabled us to unequivocally identify compounds that have toxic or beneficial effects on mitochondrial activity. Furthermore, the cross-species approach provided novel mechanistic insight and allowed early validation of hits that act on mitochondrial function.

Mitochondrial dysfunction not only typifies a range of rare inherited mitochondrial diseases^1^,^2^, but it has become clear that defects in mitochondrial function are also associated with age-related disease^3^. The concept of maintaining or repairing mitochondrial function may hence offer new preventive and therapeutic strategies for several of these rare and common diseases that still require efficient treatments.

Amongst the models that are available to explore mitochondrial defects and therapeutics, the mouse is the most widely used because of the existence of a powerful genetic toolkit and the physiological similarities to humans^4^,^5^. For example, mitochondrial defects of COX-deficient mice can be reversed using the AMPK agonist AICAR^6^. Other compounds targeting similar pathways, i.e. resveratrol and nicotinamide riboside, restore mitochondrial function in mice fed with a high-fat diet^7^,^8^,^9^,^10^. Notably, the beneficial effects of resveratrol also applied to human fatty acid oxidation-deficient fibroblasts^11^ and obese patients^12^. *C. elegans* is also used to study mitochondria, especially in the context of longevity^13^,^14^,^15^,^16^. The worm lives only weeks versus years for mice, while the ease of genetic modifications render the exploration of mitochondrial pathways accessible. Unfortunately, however, mouse and worm models are limited in throughput, precluding screening for new mitochondrial drugs^3^. One of the alternative approaches that has been developed in recent years involves phenotypic screening in both primary and immortalized cell lines^3^,^17^,^18^,^19^. These studies have shown that it is possible to identify compounds that impact on mitochondrial function without targeting a specific molecular pathway. Nevertheless, this type of approach requires a solid set of validated follow-up assays to elucidate how mitochondrial modulators work, and to narrow down several dozens of hits to compounds that are worth testing in animals.

Here, we report the development of an assay platform composed of mouse liver cells (Hepa1.6) and *C. elegans* for the systematic identification and characterization of compounds that improve mitochondrial metabolism (Figure 1). These assays are intended to assess compounds for their efficiency or toxicity on mitochondrial function. To validate our method, we first optimized cell culture conditions to ensure that the cells relied on oxidative metabolism. We next identified compounds with a strong impact on mitochondrial function out of the ~1,200 FDA-approved drugs in liver cells using a phenotypic strategy similar to that developed previously^17^. Two pharmacological classes of compounds emerged in our hit list, imidazoles and statins. We further characterized the effect of these two pharmacological classes on mitochondrial function using our set of assays. In summary, our study reports the potential of the use of an intact organism, such as *C. elegans*, in combination with mammalian cells to identify and validate hits that affect mitochondrial function at a very early stage of the drug discovery process.

## Results

### Designing a multi-level screening platform in mammalian cells and *C. elegans*

**Figure 1:**
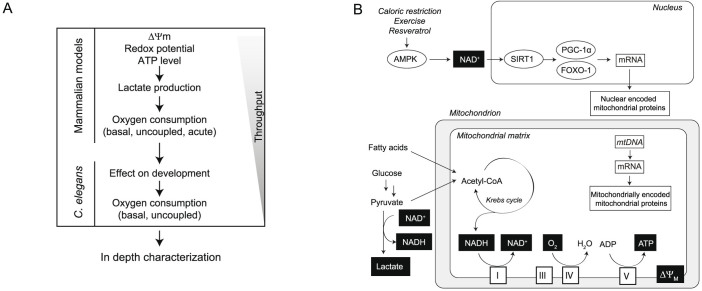
Assays platform for the identification and validation of novel mitochondrial modulators. (A) Workflow to assess mitochondrial function going from hundreds of compounds to the best candidates (top to bottom) in mammalian cells and *C. elegans*. After hits have been identified during a high-throughput screening, they are assessed for their efficiency and toxicity on mitochondrial function using this assay platform. Once the best candidates have been sorted out, they can be further investigated in both models, and ultimately in vivo. (B) The different pathways that are assessed and the respective markers that were measured (black boxes). This scheme is non-exhaustive and represents only the key regulatory steps that are surveyed. More details can be found in the main text.

We developed a platform of assays using mammalian cells and *C. elegans* to identify and validate compounds that modulate mitochondrial function (Figure 1A–B). As a starting point, we adapted a phenotypic high-throughput screening strategy that was developed and initially validated in muscle cells^17^. During the screening and validation of mitochondrial modulators, the main objective is to rapidly establish the toxicity and efficacy of the compound. Several cell culture assays were employed for this purpose. For example, measurement of ATP levels is a robust way to assess toxicity^17^,^20^ (Figure 1A–B). Metabolic redox balance, such as NAD^+^/NADH ratio (Figure 1A–B), can be measured indirectly with the use of fluorescent redox probes^21^. Nicotinamide adenine dinucleotide (NAD^+^) is not only a cofactor involved in the redox reactions, but it is also a co-substrate of the sirtuin family of deacetylases, which control mitochondrial metabolism at various levels^22^ (Figure 1B). Fluorescent probes that accumulate inside mitochondria proportionally to mitochondrial membrane potential (ΔψM) and mitochondrial abundance (e.g. tetramethylrhodamine or TMRM) can inform both about efficacy and toxicity of compounds^23^ (Figure 1A–B). Once hits are identified in this initial screen, the optimal dose for each compound can be determined in cells by using the same assays as used during high-throughput screening (Figure 1A). Mitochondrial bioenergetics, or energy flow through mitochondria, is then assessed by monitoring oxygen consumption and lactate production^18^,^19^ (Figure 1A–B). All these assays are transposable to almost every cell line, although they are representative only of the effect of a compound on the tissue from which the cell line is derived.

In this regard, *C. elegans* offers the possibility to score the impact of compounds on mitochondria and physiological homeostasis at the scale of an entire organism. A first and easy parameter to assess toxicity of a compound is to look at its impact on worm development (Figure 1A). Indeed, mitochondrial mutants were shown to have either impaired or delayed larvae growth^14^. To further characterize mitochondrial bioenergetics, oxygen consumption can also be measured in worms^13^ (Figure 1A–B).

In our model, we propose that only compounds with effects on mitochondria in both models are further validated in more expensive and time-consuming assays in both species (Figure 1A).

### Optimization of the cell culture conditions

**Figure 2:**
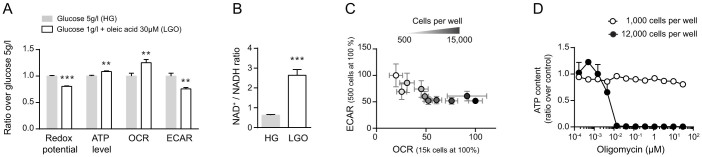
Optimization of the cell culture conditions. (A–D) Reduction in glucose concentration and supplementation with oleic acid shifts cells toward oxidative metabolism. (A) Redox potential, ATP level, oxygen consumption rate (OCR) and extra-cellular acidification rate (ECAR) measured in Hepa1.6 cells after 24 hours incubation in the indicated growth medium. (B) NAD^+^/NADH ratio determined in Hepa1.6 cells after 24 hours incubation with high glucose (5 g/l, HG); or low glucose (1 g/l) supplemented with 30 μM oleic acid (LGO) (C) Hepa1.6 cells shift toward oxidative metabolism with increasing cell confluence. OCR values are represented as a percentage of the value measured with 15,000 cells per well. ECAR values are represented as a percentage of the value measured with 500 cells per well. (D) Hepa1.6 cells are only sensitive to oligomycin at higher cell density (12,000 cells/well versus 1,000 cells/well). ATP content was normalized for each cell density over the vehicle treated control cells. For A and B, Statistical significance was determined by Student's *t*-test, with * *P*<0.05, ** *P*<0.01, *** *P*<0.001. Graphs represent mean ± SEM.

We developed our mammalian assays in the mouse hepatocyte cell line Hepa1.6, a cell line that is robust and easy to culture, and is derived from a tissue where mitochondria are abundant. We first optimized cell culture conditions to ensure that the cells solicit mitochondria to produce energy. We monitored cellular energy utilization by comparing different nutrient sources, 1) high glucose (5 g/l, HG); or 2) low glucose (1 g/l) supplemented with 30 μM oleic acid (LGO) (Figure 2A). While HG can be metabolized through glycolysis and mitochondrial oxidation, LGO forces the cells to actively rely on mitochondrial function. Indeed, LGO medium induced a marked decrease in two assays indicative of glycolysis, including redox potential, which is sensitive to NAD^+^/NADH ratio, and extra-cellular acidification rate (ECAR), a marker of lactate production (Figure 2A), In contrast, ATP level and oxygen consumption rate (OCR) were both increased in LGO medium (Figure 2A). We also observed an increase in the NAD^+^/NADH ratio in Hepa1.6 cells grown in LGO, in agreement with reduced redox potential (Figure 2B). These results show that LGO medium induces a metabolic shift from glycolysis to oxidative phosphorylation (OXPHOS).

We next tested whether cell confluence impacts on Hepa1.6 metabolism by measuring OCR and ECAR at different levels of confluence (Figure 2C). While OCR increased with cell number, ECAR decreased until a certain threshold, suggesting that Hepa1.6 cells shift from glycolysis to OXPHOS to produce ATP as confluence increases (Figure 2C). To confirm this observation, we treated Hepa1.6 cells at different cell confluences for 24 hours with the mitochondrial ATP synthase inhibitor oligomycin, and measured residual ATP levels (Figure 2D). In line with our results on the metabolic switch at higher confluence, these cells were insensitive to high doses of oligomycin at low confluence, while becoming sensitive at higher confluence (Figure 2D). Based on these results, we decided to perform all assays when the cells are at 95% confluence and using LGO as cell culture medium.

### Screening of the Prestwick library of FDA-approved drugs in Hepa1.6 cells

**Figure 3:**
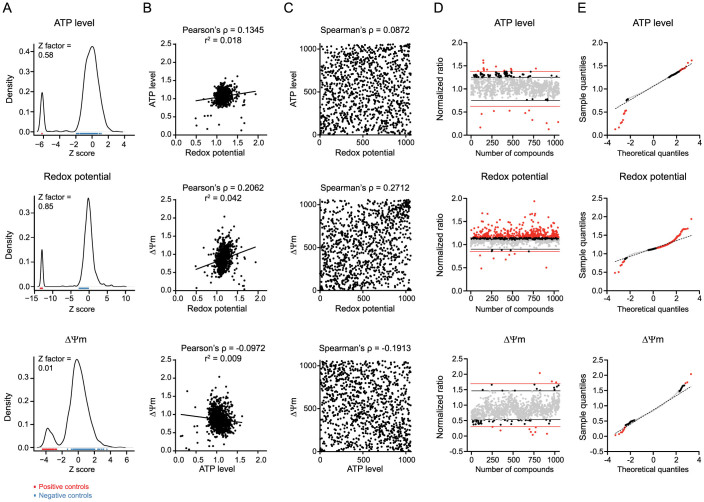
Screening of 1,200 FDA approved drugs in the Hepa1.6 cell line. (A–E) Screening of the Prestwick library of FDA approved drugs, using the three basal assays and oxidative cell culture conditions, i.e. low glucose concentration (1 g/l), supplementation with oleic acid at 30 μM and cell density of 95% the day of the assays. (A) Cell-based assays screening separation bandwidth. Plots represent the cumulative density of points over Z scores of the raw values for all the points of the screening, including negative (blue points) and positive controls (red points) and samples. The redox potential assay has the best separation capacity with narrow peaks of negative and positive controls on the density plot and a Z factor close to 1. (B–C) Correlations between the three cell-based assays show poor level of relationship between the different parameters. (B) Pearson's ρ correlation coefficients and their corresponding regression r^2^ value were calculated for normalized ratios of the three parameters. (C) Spearman's ρ correlation coefficient was calculated for the correlation between rank orders of every parameter. (D) Graphs represent the normalized ratio calculated as described in the materials and methods section. The black lines delimit the zone of compounds inducing a change inferior to DMSO ± 2σ_DMSO_, and the red lines the zone of compounds inducing a change superior to DMSO ± 3σ_DMSO_. (E) Graphs represent the quantile-quantile (QQ) plots, plotting the theoretical quantiles for each assay in x-axis over the normalized ratio in y-axis. The grey points correspond to compounds inducing a change inferior to DMSO ± 2σ_DMSO_, the black points to a change comprised between DMSO ± 2σ_DMSO_ and DMSO ± 3σ_DMSO_, and the red points to a change superior to DMSO ± 3σ_DMSO._

For the full validation of our high-throughput screen (HTS) we tested viability (ATP level), redox potential and mitochondrial membrane potential (Δψm) in Hepa1.6 cells exposed to a collection of ~1,200 mostly FDA-approved drugs in the Prestwick library (Figure 3). We obtained good separation capacity for ATP and redox potential, with a Z-factor of 0.58 and 0.85, respectively, while the Δψm assay had a Z-factor of 0.01, suggesting that this assay could potentially lead to more false positives and negatives (Figure 3A). The absence of significant correlation between the different parameters shows that the assays are complementary and non-redundant (Figure 3B–C).

**Figure 4:**
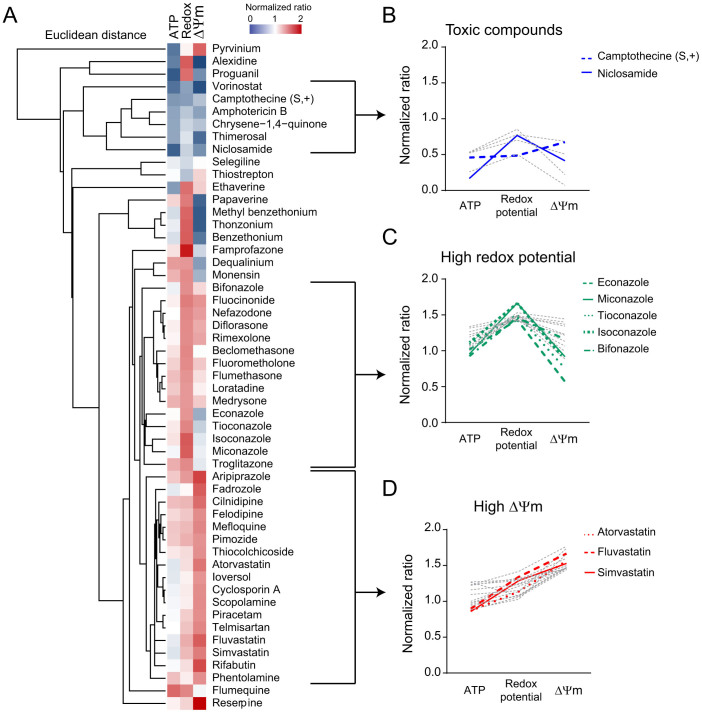
Hierarchical clustering and profiles of the 53 hits. (A) Hierarchical clustering of the 3 parameters for the 53 hits. Compounds were considered as hits when inducing a change superior to DMSO ± 2σ_DMSO_ and when deviating from normal distribution (see methods section). Heatmap represents intensities of normalized ratios. (B–D) Remarkable profiles, i.e. combinations of the scores in each of the assays. (B) Profiles of toxic compounds that reduce significantly the three parameters. (C) Profiles of the cluster of compounds that increase significantly redox potential only. (D) Profiles of the cluster of compounds that increase significantly Δψm only.

Compounds were considered as hits when inducing a signal change superior to DMSO ± 2σ_DMSO_ and when deviating from normal distribution, i.e. when squared distance between observed quantile and theoretical quantile was above a certain threshold (Figure 3D–E, methods section). 53 hits were identified after applying this two-steps selection method (Figure 4A). Hierarchical clustering was used to identify the different profiles of the hits, i.e. the combinations of the scores in each of the assays (Figure 4A). We focused our subsequent validation work on pharmacological classes that were enriched in our screen by profiles. This strategy increased our chances to characterize an effect that is due to the common mechanism of action of a pharmacological class, rather than an off-target effect of an isolated compound. One cluster contained six hits that were particularly toxic to the cells, as all three parameters decreased (Figure 4A–B). It included the antineoplastic drug camptothecine (S,+), and niclosamide, which has been reported to inhibit OXPHOS directly^24^ (Figure 4A–B). A larger cluster encompassed compounds that increased redox potential, without affecting ATP or Δψm (Figure 4A and C). Amongst these compounds, five belong to the class of antifungal imidazoles (Figure 4A and C). Imidazoles block demethylation of lanosterol through the inhibition of sterol 14α-demethylase (CYP51A1)^25^,^26^, an essential step in the synthesis of ergosterol in fungi and cholesterol in mammals. Other reports show that their antifungal effect is also mediated by an induction of uncoupling and swelling of rat liver mitochondria^27^. Imidazoles henceforth represent a class of compounds that are potentially toxic to the mitochondria. A third remarkable cluster regrouped compounds that induce Δψm, without affecting ATP or redox potential (Figure 4A and D). Strikingly, this set of Δψm inducers contained three statins (Figure 4A and D). Statins inhibit one of the initial steps in cholesterol biosynthesis, but this also affects the synthesis of coenzyme Q10 (CoQ10), heme-A and substrates for protein prenylation^28^. Therefore, statins represent a class of compounds that may also induce mitochondrial stress, but have an overall demonstrated beneficial impact on human health and longevity^29^.

Considering the overrepresentation of statins and imidazoles in our hit list, we decided to further characterize their effect on mitochondrial function using our mammalian and *C. elegans* assays (Figure 1A).

### Impact of imidazoles on metabolism in cultured cells and *C. elegans*

**Figure 5:**
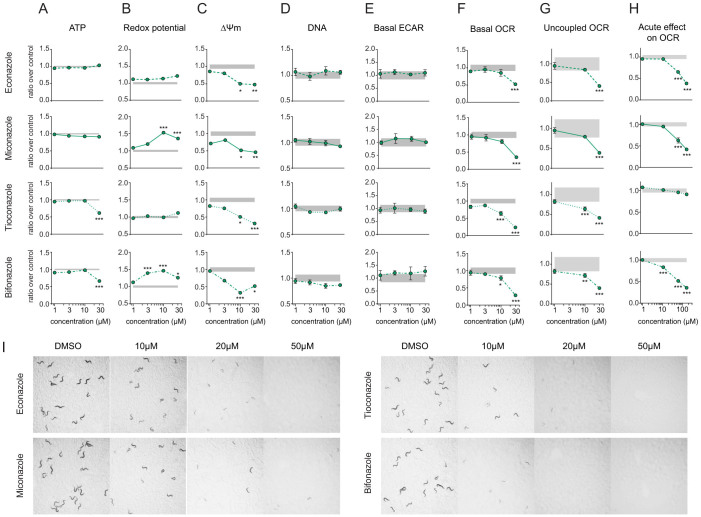
Impact of imidazoles on cellular and *C. elegans* metabolism. (A–G) Effect of 24 hours treatment with econazole, miconazole, tioconazole and bifonazole in Hepa1.6 on ATP level (A), redox potential (B), Δψm (C), DNA content (D), extra-cellular acidification rate (ECAR) (E), basal oxygen consumption rate (OCR) (F) and uncoupled OCR (G). (H) Acute effect of imidazoles on OCR in Hepa1.6. Measurement was performed 3 minutes after addition of the compounds. Results are expressed as ratio over DMSO control and as mean ± SEM. Grey zone represents the DMSO 95% confidence interval. Significance was tested using a two-way ANOVA test, with Bonferroni correction for multiple comparison. * *P*<0.05, ** *P*<0.01 and *** *P*<0.001. Graphs represent mean ± SEM. (I) Econazole, miconazole, tioconazole and bifonazole impair the development of *C. elegans* N2 worms at all tested concentrations. Pictures were taken at day 3 of adulthood.

We first performed dose-response tests for all our mammalian assays using the four imidazoles identified, i.e. econazole, miconazole, tioconazole and bifonazole (Figure 5A–H). Tioconazole and bifonazole showed toxic effects on ATP levels, but only at 30 μM (Figure 5A). Miconazole and bifonazole increased redox potential by up to 50% at 10 and 30 μM, while econazole and tioconazole gave milder responses (Figure 5B). All four imidazoles decreased Δψm by at least 50% at 10 and 30 μM (Figure 5C). The reason why these compounds did not decrease Δψm significantly during the HTS (Figure 4A and C) is explained by the high variability of this assay, leading to a higher threshold of significance when testing multiple samples. All these changes were independent on cell number, as assessed by DNA quantification (Figure 5D). Long-term treatment of Hepa1.6 (24 hours) cells with imidazoles induced a metabolic shift toward glycolysis, as ECAR was maintained (Figure 5E) while basal OCR significantly decreased (Figure 5F). Uncoupled OCR was also significantly reduced after imidazoles treatment, indicating a decrease in maximal respiratory capacity (Figure 5G). Measurement of OCR after short-term treatment showed that econazole, miconazole and bifonazole inhibit cellular respiration within a few minutes (Figure 5H), suggesting that imidazoles inhibit the mitochondria directly and acutely. This toxic effect of imidazoles was confirmed in *C. elegans*, as they all strongly disturbed worm development at 10 μM and higher doses (Figure 5I).

The fact that *C. elegans* are cholesterol auxotrophs and are supplemented with cholesterol during the experiments^27^,^30^,^31^ underscores that the imidazole toxicity is independent of cholesterol depletion. Instead, imidazoles have an acute inhibitory effect on respiration, in line with previous reports^27^. A potential explanation for this effect is the inhibition of the transient receptor potential cation channel, subfamily M, member 2 (TRPM2), a calcium-permeable cation channel that is regulated by free intracellular ADP-ribose^32^. Importantly, even though the imidazoles are toxic in our experimental setup, they are mostly used topically in the clinic. In such application, less than 1% reaches the systemic circulation^33^, which explains why this is unlikely to cause major systemic side effects.

### Impact of statins on metabolism in cultured cells and *C. elegans*

**Figure 6:**
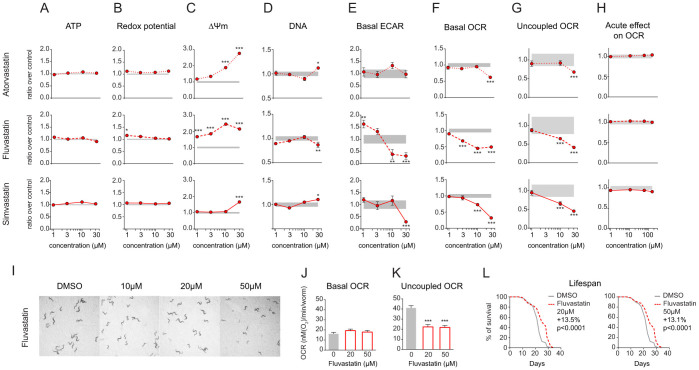
Impact of statins on cellular and *C. elegans* metabolism. (A–G) Effect of 24 hours treatment with atorvastatin, fluvastatin and simvastatin in Hepa1.6 on ATP level (A), redox potential (B), Δψm (C), DNA content (D), extra-cellular acidification rate (ECAR) (E), basal oxygen consumption rate (OCR) (F) and uncoupled OCR (G). (H) Acute effect of statins on OCR in Hepa1.6. Measurement was performed 3 minutes after addition of the compounds. (I) Fluvastatin does not impair the development of *C. elegans* N2 worms at 10, 20 and 50 μM. Pictures were taken at day 3 of adulthood. (J–K) Effect of fluvastatin on basal OCR (J) and uncoupled OCR (K) at day 1 of adulthood. (L) Lifespan in N2 worms after treatment with fluvastatin at 20 and 50 μM. Results are expressed as ratio over DMSO control and as mean ± SEM. Grey zone represents the DMSO 95% confidence interval. Significance was tested using a two-way ANOVA test, with Bonferroni correction for multiple comparison. * *P*<0.05, ** *P*<0.01 and *** *P*<0.001.

Similar to our strategy for imidazoles, we first performed dose-response studies for redox potential, ATP level and Δψm using the different statins (Figure 6A–C). All three statins induced Δψm by up to 3-fold in Hepa1.6 cells, indicating a strong hyperpolarization, while ATP level and redox potential did not change (Figure 6A–C). The effect on Δψm was not due to an increase in the number of cells, which was only mildly increased at the highest dose with atorvastatin and simvastatin (Figure 6D). Treatment of Hepa1.6 cells for 24 hours with fluvastatin and simvastatin decreased both OCR and ECAR, but only OCR with atorvastatin (Figure 6E–F), suggesting an overall decrease in metabolic rate of the cells. The decrease in OCR was maintained when OXPHOS was uncoupled (Figure 6G), indicating a decrease in overall respiratory capacity. However, unlike imidazoles, statins did not inhibit respiration in Hepa1.6 cells acutely (Figure 6H).

The effect of statins was further investigated in *C. elegans* using fluvastatin. This statin was selected as simvastatin and atorvastatin, but not fluvastatin, crystallized at 20 μM in the agar plates used for worm culture. Although high doses of statins (mM) lead to embryonic lethality in worms due to the impaired prenylation of small GTPases^34^, we found that fluvastatin did not impair larval development up to the concentration of 50 μM (Figure 6I). We then tested the effect of fluvastatin on worm respiration (Figure 6J–K). While basal OCR was similar to untreated worms, addition of FCCP did not increase OCR in fluvastatin-treated worms (Figure 6J–K), suggesting that the impact of statins on respiration was conserved through the different models. Finally, fluvastatin increased worm mean lifespan by 14% at 20 μM and by 13% at 50 μM (Figure 6L), indicating that the decrease in respiration induced by fluvastatin has an overall beneficial effect in worms.

**Figure 7:**
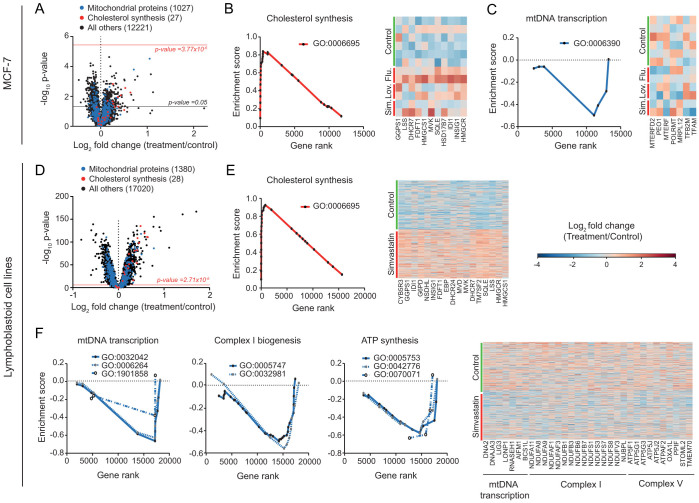
Impact of simvastatin on gene expression in MCF-7 cells and human lymphoblastoid cell lines. (A–C) Effect of three statins, fluvastatin (Flu.), lovastatin (Lov.) and simvastatin (Sim.), on gene expression in MCF-7 cells after 6 hours treatment at 10 μM. (A) Vulcano plot of microarray dataset. Treatment/control fold change expression was determined by calculating first the average expression for all statins and DMSO treatments. Cholesterol synthesis geneset (GO:0006695) and mitochondria geneset (GO:0005739) are represented in red and blue, respectively. Black horizontal bar corresponds to the significance threshold for a nominal p-value of 0.05, while the red horizontal bar corresponds to the p-value for multiple testing after Bonferroni correction. (B) Positive enrichment score indicates that MCF-7 cells induce cholesterol synthesis genes (geneset GO:0006695, p<0.01) following sterol depletion. (C) Negative enrichment score for mtDNA transcription genes (geneset GO:0006390, q = 0.24) shows that mtDNA processing is downregulated after statins treatment in MCF-7 cells. (D–F) Effect of simvastatin on gene expression in lymphoblastoid cell lines derived from patients after 24 hours treatment at 3 μM. (D) Vulcano plot of microarray dataset. Cholesterol synthesis geneset (GO:0006695) and mitochondria geneset (GO:0005739) are represented in red and blue, respectively. The red horizontal bar corresponds to the p-value for multiple testing after Bonferroni correction. (E) Positive enrichment score indicates that in lymphoblastoid cells simvastatin induces cholesterol synthesis genes (geneset GO:0006695, p<0.001). (F) Negative enrichment scores for mtDNA transcription genes (genesets GO:0006264, q = 0.009; GO:0032042, q = 0.003 and GO:1901858, q = 0.084), respiratory complex I (genesets GO:0005747, q = 0.052 and GO:0032981, q = 0.143) and respiratory complex V (genesets GO:005753, q = 0.015; GO:0042776, q = 0.039 and GO:0070071, q = 0.079) show that respiratory complexes and mtDNA processing is downregulated after simvastatin treatment in lymphoblastoid cells. For B, C, E and F, genesets were determined according to Gene Ontology annotation (www.geneontology.org). Heatmaps represented in E and F show only the genes that are significantly differentially expressed between control (green bar) and statin (red bar) treated samples according to nominal p values.

A potential mechanism to explain the decreased respiratory capacity in cells and worms after long-term statin treatment is the inhibition of mevalonate synthesis, which in turn limits CoQ10 and heme-A levels^28^, two essential components of the mitochondrial respiratory chain^35^. Another possibility is that these effects are mediated at the transcriptional level. To test this hypothesis, we analyzed a dataset from the Connectivity Map database (http://lincs.hms.harvard.edu/db/). This data set contains gene expression profiles of the human breast cancer cell line MCF-7 exposed for 6 hours to three different statins, i.e. fluvastatin, simvastatin and lovastatin at 10 μM. The change in gene expression after statin treatment was low because of the limited number of samples per condition (Figure 7A). As expected, genes involved in cholesterol biosynthesis were strongly upregulated (Figure 7B). Additionally, we noted a significant downregulation of genes involved in mtDNA transcription (Figure 7C).

As a further translation of our data towards human relevance, we performed a gene-set enrichment analysis on gene expression data from 480 patient-derived lymphoblastoid cell lines that were exposed for 24 hours to vehicle or 3 μM simvastatin^36^. The overall gene expression variation in these cells after simvastatin treatment was low, but due to the high number of samples per conditions, 26% of genes presented a significant change (Figure 7D). Similar to the Connectivity Map data, the cholesterol biosynthesis pathway was strongly induced by simvastatin (Figure 7E)^36^,^37^, and several mitochondrial gene sets related to mtDNA transcription were downregulated (Figure 7F). In addition, expression of genes related to respiratory complex I and V were also repressed (Figure 7F). These data suggest that the mitochondrial functional changes we observed in mouse hepatocytes and worms are conserved in humans and are common to at least fluvastatin, simvastatin and lovastatin.

## Discussion

We developed a set of straight-forward assays in two different model systems to rapidly identify whether a compound may have potential therapeutic benefits at the level of mitochondria.

The first model is an immortalized hepatic cell line, Hepa1.6. Immortalized cell lines offer the advantage that they can be grown in large quantities and high number of passages, two features that make their use easier when working at a high-throughput level. However, the immortalization process is accompanied by metabolic changes similar to those observed in cancer cell lines, including the presence of the so-called Warburg effect^38^. A careful optimization of cell culture conditions is therefore required to ensure that the cells rely on mitochondrial metabolism for energy production. Supplementing oleic acid in the culture medium and increasing cell confluence stimulated Hepa1.6 cells to switch from glycolytic to mitochondrial metabolism (Figure 2). In this cell line, we measured several parameters that displayed a wide dynamic range during our screening. This allowed the identification of both activators and inhibitors of mitochondrial function. In fact, we were able to stratify the hits according to their response and divided into them separate classes, i.e. compounds toxic to mitochondria and cells (e.g. papaverine, niclosamide), compounds that induce redox potential (imidazoles) and those that induce Δψm signal (statins).

Hits from our high-throughput screening were selected for the evaluation of respiration and lactate production. The increase in the throughput of the respiration assays enabled the measurement of up to four parameters related to catabolism (ECAR, basal OCR and uncoupled OCR after long-term treatment, and OCR after acute treatment). Notably, the determination of OCR after acute incubation with the compounds allowed us to distinguish between the direct impact of imidazoles on mitochondria versus the indirect effect of the statins on respiration, only evident after long-term treatment.

The second model that we used to analyze mitochondrial function was the roundworm *C. elegans*. It has the advantage to provide insights on the effects of compounds in an intact animal and at the scale of multiple tissues. One of our read-outs was the impact of compounds on larval development. *C.elegans* development depends on the accurate timing of cell division and differentiation, which requires a complex combination of genetic and molecular mechanisms^39^. In particular, development of *C.elegans* is highly energy-dependent and relies on the electron transport chain (ETC), as it was shown that food deprivation and downregulation of ETC complexes expression strongly delayed or stopped the larval development^40^. In line with these premises, imidazoles, but not statins, caused a severe developmental delay. Since *C. elegans* are cholesterol auxotrophs, our data suggest that the toxicity of the imidazoles is independent of cholesterol depletion. Instead, imidazoles have an acute inhibitory effect on respiration, which may be due to the induction of uncoupling and swelling of mitochondria, as previously documented in isolated rat liver mitochondria exposed to econazole^27^.

In contrast, the mechanism by which statins affect mitochondrial respiration was not associated with toxicity, as Hepa1.6 cells were able to maintain their ATP and redox potential levels, and worm development was not impacted. Our result hence contrasts with previous reports showing that high pharmacological doses of statins inhibit *C. elegans* development^34^. We used, however, at least ten times lower doses, and stayed below doses at which the hydrophobic fluvastatin crystallizes in agar plates. Working in these more gentle and physiological conditions, we were able to show for the first time that fluvastatin increased worm lifespan, in line with the lifespan extension observed in *Drosophila* and the reduction in all-cause mortality measured in humans after simvastatin treatment^41^,^42^,^43^,^44^. Remarkably, the fact that statins extend lifespan in lower organisms suggests that their therapeutic benefit in human may also be partly independent of their effect on cholesterol biosynthesis.

Two potential mechanisms can explain the decreased respiratory capacity in cells and worms after long-term statin treatment. First, by inhibiting mevalonate synthesis, statins also impact on the levels of CoQ10 and heme-A^28^, which are both essential for respiratory chain function^35^. Second, gene expression data from both lymphoblastoid cell lines and MCF-7 cells show that there is also a downregulation of genes involved in mtDNA transcription. In the lymphoblastoid cell lines, there is furthermore a pronounced decrease in the expression of genes encoding for components of complex I and V subunits. The link between these data and the increase in Δψm after statins is less clear. In fact, Δψ_m_ is decreased in cells with defects in respiratory complexes I and II^45^,^46^. However, another study showed that in neurons deficient for respiratory complex I, Δψ_m_ was increased because ATP synthase is working in reverse mode^47^. Overall, these data underline the complexity of the metabolic remodeling induced by statins treatment. This complexity is directly linked to the number of pathways affected by the inhibition of mevalonate synthesis, which is an early precursor of multiple metabolites.

In conclusion, we developed a cross-species mitochondrial screening and validation platform, using both mammalian cell lines and *C. elegans*, exploiting and emphasizing the strengths of each of these models. Using this screen, we identified negative and positive regulators of mitochondrial function. The combination of multiple tests that inform about distinct aspects of mitochondria enabled us to define a complex and comprehensive signature of the actions of pharmacological compounds on mitochondria. This setup allowed us to define the effects of imidazoles and statins on mitochondrial function, and identify potential novel pathways that may contribute to the pleiotropic effects observed upon statin treatment. We envision that such a comprehensive screening approach is essential to identify novel mitochondrial modulators. Such compounds will be beneficial for both rare and common diseases in which mitochondria are dysfunctional.

## Methods

### Compounds used in cell-based assays and *C. elegans*

The Prestwick Chemical Library, which contains 1,200 small molecules and FDA approved drugs, was acquired at Prestwick Chemicals (Illkirch-Graffenstaden, France). Fluvastatin, simvastatin and atorvastatin were provided by Prestwick Chemicals (Illkirch-Graffenstaden, France). Econazole, miconazole, tioconazole and bifonazole were acquired at Sigma (Sigma-Aldrich, St. Louis, MO, USA).

### Cell lines and medium conditions

Hepa1.6 mouse hepatoma cells were obtained from ATCC and maintained according to ATCC guidelines. For the assays, hepa1.6 cells were plated in low glucose medium (1 g/l glucose) supplemented with 30 μM oleate-BSA (LGO) (Sigma-Aldrich, St. Louis, MO, USA), unless specified otherwise.

### High-throughput cell-based assays

Hepa1.6 cells were seeded at 8,000 cells per well in a volume of 15 μl, in 384-well plates. Compounds and were added from a 4x concentrated stock, diluted in culture medium, to the cells, reaching a total assay volume of 20 μl. Assays were typically performed 24 hours after incubation with medium or compounds. ATP levels were measured using Cell Titer Glo (Promega, Madison, WI, USA). Mitochondrial membrane potential (Δψm) was measured using the fluorescent tetramethylrhodamine methyl ester (TMRM) probe (T-668, Invitrogen, Paisley, UK). Briefly, cells were incubated in culture medium and TMRM was added to the culture medium at a final concentration of 200 nM for 45 minutes at 37°C before washing with 50 μl of PBS. The Alamar Blue assay (Life Technologies, Paisley, UK) was used as a marker for redox potential, following manufacturer protocols*.* ATP level, redox potential and TMRM assays were measured on a Tecan Infinite 500 (Tecan, Männedorf, Switzerland), and performed in duplicate.

### Screening of a 1,200 drugs library - specific procedures

Hepa1.6 cells were seeded at 8,000 cells per well, in a volume of 20 μl, in 384-well plates, treated on day 1 using a Sciclone G3 Liquid Handling Workstation (PerkinElmer, Waltham, MA, USA) and distributing a volume of 2.22 μl of compounds at 100 μM to obtain a final concentration of 10 μM in each well. Assays were performed after 24 hours of incubation with compounds. For ATP level, redox potential and TMRM assays, we used DMSO at 0.1% as a negative control (final DMSO concentration in all wells) and oligomycin A at 1 μM as a positive control, both of them being present on each side of all the plates, i.e. columns 23–24 and 1–2 respectively. These assays were performed in duplicate.

High-throughput screening data were analyzed using the cellHTS2 package from R software (http://www.r-project.org/). ATP, redox potential and TMRM, data were normalized per plate using the normalized percentage of inhibition function (normalized ratio), calculated as follows: 
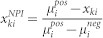
, where *x_ki_* is a result point, 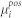 is the average for all the oligomycin A positive control points and 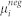 is the average of the DMSO negative control points. Z scores and Z factors were calculated from these normalized values for the Figure 3A. Theoretical quantiles (Figure 3E) were calculated from the normalized values using R software. Compounds were considered as hits when inducing a signal change superior to DMSO ± 2σ_DMSO_ and when deviating from normal distribution, i.e. when squared distance between observed quantile and theoretical quantile was above a certain threshold. This threshold was arbitrarily set at 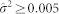 for ATP, TMRM and redox potential signals. Clustering of the 53 hits was performed on the Hierarchical Clustering module available on the GenePattern website (http://www.broadinstitute.org/cancer/software/genepattern), using Euclidean distance and single linkage as analysis parameters.

### Measurement of cellular NAD^+^ and NADH levels

NAD^+^ and NADH levels were determined using the EnzyChrom™ NAD/NADH Assay kit and following manufacturer's instructions (BioAssays Systems, Hayward, CA, USA)

### Measurement of DNA levels per cell

DNA was measured directly in Seahorse 96-well plates using the CyQUANT® Cell Proliferation Assay Kit and following manufacturer's intructions (Lifetechnologies, ThermoFisher Scientific, Waltham, MA, USA).

### Measurement of cellular and *C. elegans* respiration

Oxygen consumption rate (OCR) and extra-cellular acidification rate (ECAR) were measured using the Seahorse XF96 equipment (Seahorse bioscience Inc., North Billerica, MA, USA). For measurement of cellular metabolism, Hepa1.6 were seeded at 12,000 cells per well and treated with different medium conditions or compounds for 24 hours in 150 μl medium. Acute effect of compounds was evaluated by injecting them during the run. Worm oxygen consumption was performed as described^48^. Briefly, at day 1 of adulthood, ten worms per well were transferred into M9-filled 96-well Seahorse plates. Oxygen consumption was measured 6 times in basal conditions. FCCP treatment was performed at 10 μM final.

### Pharmacological treatment of *C. elegans* strains

*C. elegans* strains were cultured at 20°C on nematode growth media agar plates seeded with *E. coli* strain HT115 (DE3). Strains used were wild-type Bristol N2 and provided by the Caenorhabditis Genetics Center (University of Minnesota). Imidazoles and statins were added at the indicated concentrations just before pouring the plates. Animals were exposed to compounds during the full life from hatching onwards. To ensure a permanent exposure to the compound, plates were changed twice a week for the lifespan assays. Imidazoles and statins were dissolved in DMSO, and the vehicle controls were adapted accordingly.

### Lifespan and development assays

Lifespan tests were performed as described at 20°C^49^. Briefly, 60–100 animals were used per conditions and scored every other day. Animals that crawled off the plate or had an «exploded vulva» phenotype were censored. The development of a synchronized worm population was monitored after 4 days of post-embryonic development by taking pictures of the whole plate.

### Worm statistics

Survival analyses were performed using the Kaplan Meier method and the significance of differences between survival curves calculated using the log rank test. Calculation of mean lifespan and SEM were calculated using the R software.

### Bioinformatics analysis

The datasets generated in human lymphoblastoid cell lines^36^ was downloaded from Gene Expression Omnibus under the accession number GSE36868. The datasets generated in the human breast cancer cell line MCF-7 was downloaded from the Connectivity Map database (http://lincs.hms.harvard.edu/db/). Data were first normalized with RMAExpress software (http://rmaexpress.bmbolstad.com/) and analyzed using the Gene Set Enrichment Analysis software^50^. The cholesterol synthesis geneset was defined according to GO annotation (Accession number: 0006695). In this case, since a single gene set was tested (e.g. cholesterol gene set), a nominal p value inferior to 0.05 was used as significance threshold. Mitochondrial genesets were defined by searching for all gene ontologies containing the word “mitochondria*” on AmiGO website (http://amigo.geneontology.org/cgi-bin/amigo/go.cgi). A total of 121 genesets were defined using this method. In this case, several genesets were tested and the q value of the false discovery rate control inferior to 0.25 was used as a significance threshold.

## Author Contributions

P.A., R.H. and J.A. conceived the project. P.A., L.M., R.H. and J.A. wrote the manuscript and J.A. supervised the project. P.A., S.B. and N.M. performed high throughput screening and validation experiments in vitro. P.A. performed high-throughput screening data analysis. P.A., L.M., S.B., V.J. and A.M. performed *C. elegans* experiments. P.A. and X.W. performed bioinformatics analysis.
